# Necrology

**Published:** 1900-07

**Authors:** 


### NECROLOGY.

Charles A. Mackey, V.S., of New York City, died at his residence in April. The deceased was a graduate of the New York College of Veterinary Surgeons, class of 1889.

After a long and painful illness, the wife of Dr. William Jopling, of Owosso, Mich., passed peacefully away in March last.

Dr. Jopling's work as President of the State Association is well known, though for more than a year restricted by the exactions of the continued illness of his wife.

